# Recurrent Chest Pain, as a Presenting Sign of Ovarian Endometrioma

**DOI:** 10.5402/2011/837501

**Published:** 2011-05-02

**Authors:** Mehmet Yildirim, Ozgur Oztekin, Deniz Oztekin

**Affiliations:** ^1^Department of Surgery, Izmir Bozyaka Teaching and Research Hospital, 35540 Izmir, Turkey; ^2^Department of Radiology, Izmir Bozyaka Teaching and Research Hospital, Izmir, Turkey; ^3^Department of Obstetrics and Gynecology, Aegean Obstetrics and Gynecology Training and Research Hospital, Izmir, Turkey

## Abstract

Chest pain is a rare sign of thoracal endometriosis associated with endometrioma of the tubo-ovarian endometrioma. We report the case periodic episodes of chest pain concurrent with menstruation in a 35-year-old female, in which ovarian endometrioma was diagnosed and left-sided oophorectomy was performed. After surgery, patient underwent medical treatment which included a Gn-RH agonist and a combined oral contraceptive. In the follow-up period, there was no evidence of chest pain.

## 1. Introduction

Endometriosis is a common gynecologic disorder affecting women during their reproductive years [1]. Pathologically, it is the result of functional endometrium located outside the uterus. The ovaries are the most common sites affected, but endometriosis can also involve the gastrointestinal tract, urinary tract, chest, and soft tissues [2]. Endometriosis of the chest is uncommon, and the diagnosis is usually established on clinical grounds. Endometrial tissue may involve the pleura by migrating from the peritoneal cavity to the pleural cavity through diaphragmatic defects or via microembolization. It is estimated that nearly 2% of cases of extrapelvic endometriosis involve the thorax. Pleural lesions are almost exclusively rightsided. Pleuritic chest pain, pneumothorax, pleural effusions, or cyclic haemoptysis can occur with pulmonary involvement [3–5].

## 2. Case Report

A 35-year-old female presented with episodes of a right chest pain approximately 3-4 days in a month and resolved spontaneously. Her primary care physician had treated her with antibiotics empirically throughout the four months. During the last episode of chest pain, the patient described having a pain not resolving with analgesics combination. The patient did not have any exacerbating factors, fevers, dyspnoea, haemoptysis, weight loss, and gastrointestinal complaints. 

The patient's medical history was unremarkable, and she specifically denied a history of trauma and cardiopulmonary disease. She had spent 4 months in seaside town and had also worked at a seafood restaurant. She denied use of alcohol but not smoking. Her physical examination was within normal limits, except minimal pain in the right-sided lower chest and tenderness of the left lower abdominal quadrant. Routine laboratory tests and tumor markers (AFP, CEA, CA 15-3, CA 19-9) were within normal limits but increased serum level of CA-125 level (53.7 U/mL, normal value: 0–35 U/mL).

**Figure 1:**
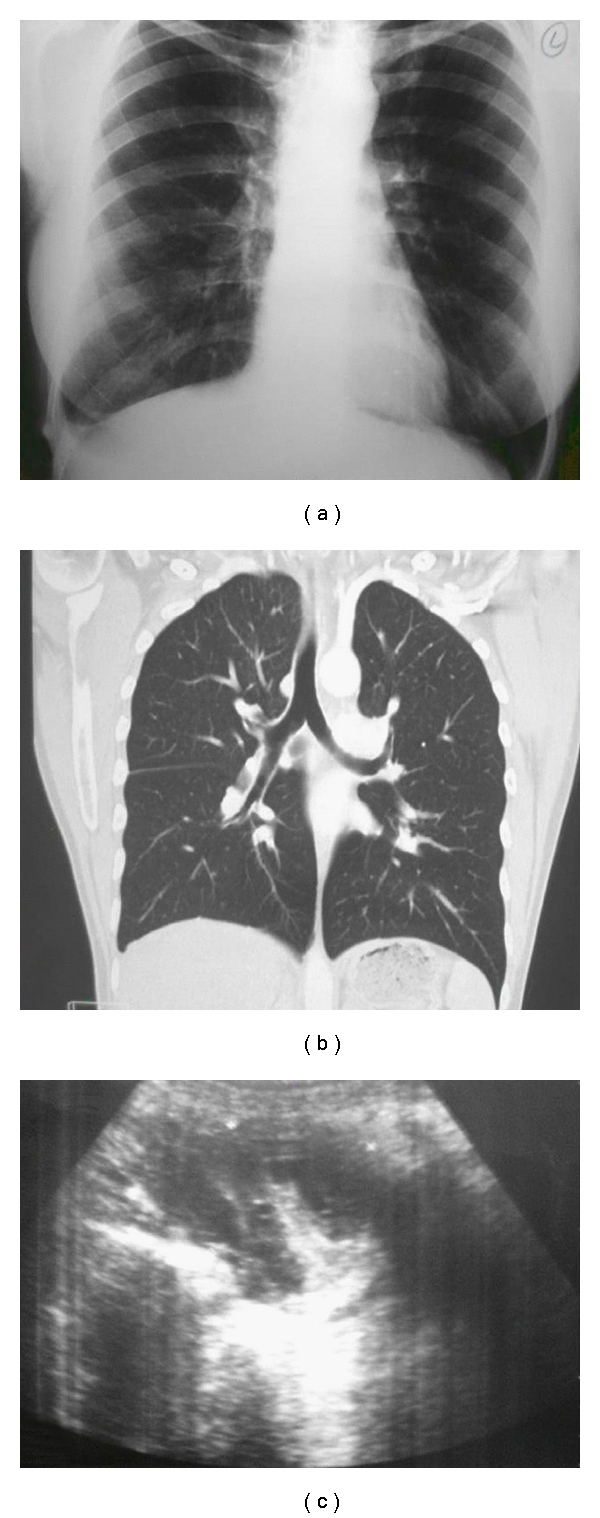
(a) Chest radiography showed a closed right-sided costaphrenic angle and minimal pleural effusion. (b) CT scan did not demonstrate any abnormality. (c) Pelvic US revealed a left adnexal semisolid mass, with a thick wall and septa.

Her chest radiography showed a closed right-sided costophrenic angle and minimal pleural effusion without evidence of pneumonia (Figure 1(a)). Chest computed, tomography (CT) scans were performed 6 days after the onset of menstruation, and these did not demonstrate pleural effusion or any abnormality (Figure 1(b)). No abnormalities were noted after consultation by chest physicians.

**Figure 2:**
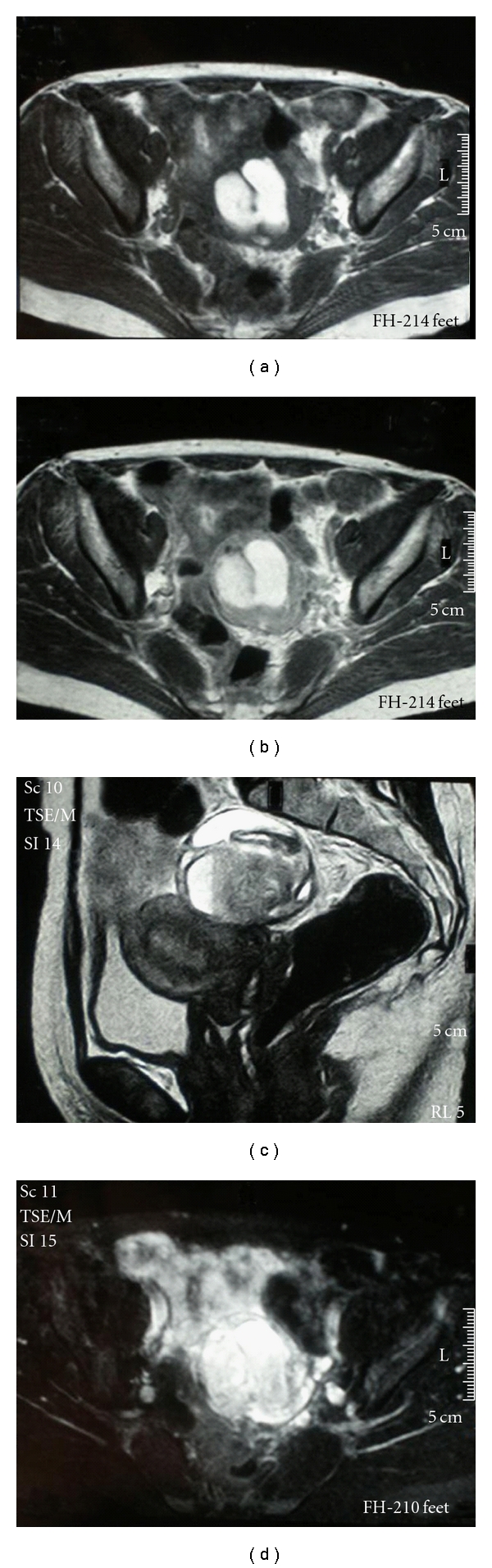
(a-b) T1-weighted MR image shows a unilocular high-signalintensity mass on the left side. (c-d) T2-weighted image shows that the left-sided mass remains high in signal intensity. The variable appearance of the lesion is thought to reflect the blood products.

A percutane thoracal punction (no abnormalities were noted on histopathologic examination) was done because she denied thoracoscopic examination. Pelvic endometriosis was considered a possible diagnosis according to the results of US and MR of the abdomen. Pelvic US revealed a left adnexal semisolid mass, with a thick wall and septa, measuring 71 × 61 cm (Figure 1(c)). MR imaging evaluated a left adnexal mass in 67 × 57 mm diameter with, high-signalintensity areas on T1- (Figures 2(a) and 2(b)) and T2-weighted (Figures 2(c) and 2(d)) images. In addition, dynamic signal range was not narrowed with fat-suppressed sequence, and heterogeneous pattern was found on postcontrast series. Therefore, based on imaging characteristics immature teratoma, endothermal sinus tumor, and germ cell tumor must be considered primarily. 

**Figure 3:**
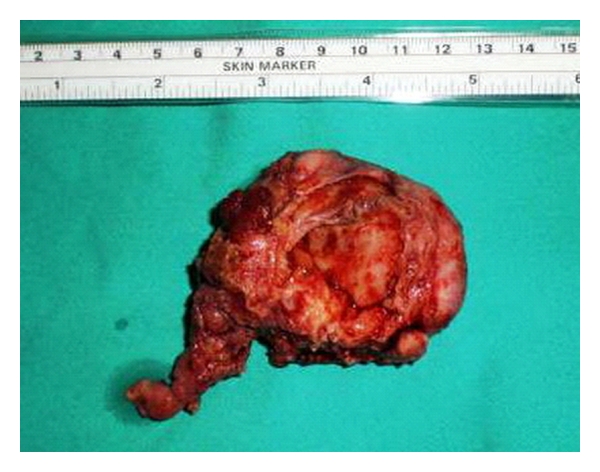
Photograph of the gross specimen reveals multiple dark-brown, hemorrhagic foci and irregular cysts in the internal surface.

At exploratory laparotomy, a transverse incision was performed, and a 6 cm mass was found just behind the uterus, as well as adhesions between the mass and the adjacent tissues. The patient needed sharp dissection and underwent adhesiolysis with left-sided salpingo-oophorectomy. Rest of endometriotic focuses on uterus, pouch of Douglas, and other peritoneal surfaces were destroyed with electro-coagulation. Macroscopic appearance showed multiple irregular hemorrhagic foci and chocolate cysts (Figure 3). 

Histopathological examination of the mass revealed tuba-ovarian endometriosis. The postoperative recovery was uneventful, and the patient was discharged on the third post-operative day. Leuprolide acetate (Lucrin 3.75 mg; Abbott Australasia, Kurnell, NSW, Australia) was used to achieve endometrial atrophy for six months after surgery. Medical treatment was continued with a cyclic low-dose combined oral contraceptive (Yasmin; Bayer HealthCare Pharmaceuticals Inc., Wayne, NJy). One year of regular followup, there was no evidence of recurrent pleural effusion, chest pain, and abdominal complaints.

## 3. Discussion

Endometriosis is a complex disorder, and its causes are probably multifactorial. The theory stated that endometriosis results from transportation of endometrial tissue from retrograde menstruation. The endometrial cells implant on serosal surfaces into the peritoneal cavity and transport to the thorax through diaphragmatic defects and lymphatic or vascular channels. 

Thoracal endometriosis, depending on the extent and tissue affected, can produce pleuritic chest pain, pleural effusion, hemothorax, and pneumothorax. The physical manifestations are variable, with some patients being asymptomatic and others having disabling chest or pelvic pain, adnexal masses, or unusually signs. In our patient, periodic episodes of symptoms concurrent with menstruation led to the suspicion of a relationship between these conditions. The peak incidence for pelvic endometriosis occurred between 24 and 29 years, whereas the peak incidence for thoracal involvement was between 30 and 34 years [6]. In a series of 110 patients with thoracal endometriosis, pneumothorax, hemothorax, hemoptysis, and pulmonary nodules were found in 73%, 14%, 7%, and in 6% patients [6]. The right hemithorax was involved in more than 90% of all manifestations except for nodules [7]. Regardless of pathophysiology, thoracal endometriosis is generally associated with coexistent pelvic endometriosis and usually occurs 5 years after the diagnosis of pelvic endometriosis. However, most women with endometriosis have normal or nonspecific results from physical examinations, and laparoscopy is necessary for the definitive diagnosis. For the definite confirmation of presence of endometrial tissue CT-guided percutaneous transthoracic needle biopsy [8] or thoracoscopic tissue biopsy should be performed. By immunohistochemical analysis oestrogen, progesterone and CD10 receptors can be shown in the biopsy material. We only able to perform thoracal CT and abdominal MR imaging in present case, because our patient denied a thoracoscopic examination.

The US appearance of pelvic endometriosis exhibits diffuse low-level internal echoes, hypoechoic focal lesion, and rarely, may be anechoic, mimicking a functional ovarian cyst. Patel et al. found that wall thickness is not a differentiating feature between endometriomas and other ovarian masses [9]. Using CT, endometriosis of the lung parenchyma can be determined particularly during menstruation with unifocal or multiple nodulr lesions. MR imaging has been shown to have greater specificity for the diagnosis of abdominal endometriomas. A study confirmed a left lateral predisposition of endometrioma [10]. Endometriomas have a relatively homogeneous high-signalintensity on T1-weighted images. Lesions with degenerated blood products, including concentrated protein, appear with high-signalintensity areas on T1- and T2-weighted images. In our case, T1-weighted MR image showed a multilocular high-signalintensity mass on the left ovary, and highness in signal intensity remained in T2-weighted image. A common feature of an endometrioma, shading, is present when a cyst that is hyperintense on a T1-weighted image becomes hypointense on a T2-weighted image. This shading reflects the chronic nature of an endometrioma and helps differentiate it from other blood-containing lesions except hemorrhagic corpus luteum cysts, which do not exhibit shading on T2-weighted images [1]. Other lesions that appear with high-signalintensity on T1-weighted images include dermoids, mucinous cystic tumors, and hemorrhagic masses. 

Treatment for thoracal endometriosis can be medical for chest pain and effusion or surgical, depending on the severity of urgent and elective symptoms. Surgical pleurodesis result in low recurrence rate for pneumothorax or hemothorax. Medical treatment is one of the choices of treatment to relieve cyclic pain in endometriosis. Multiple pharmacologic agents in use include combined oral contraceptives, danazol, Gn-RH agonists and progestins [2]. In our patient, medical treatment with a Gn-RH agonist, which was continued with a cyclic low-dose combined oral contraseptive was effective to relieve cyclic pain. Definitive surgery includes hysterectomy and oophorectomy and is usually reserved for women with intractable pain. The conservative surgery, including left-sided oophorectomy, was performed to our patient.

In our patient, chest pain helped to diagnose the tubo-ovarian endometrioma. In our case, we did not establish pleural endometriosis cytologically but the monthly chest pain associated with endometrioma of the ovary supported extrapelvic lesion, clinically.
